# A meta-analysis of the association between vitamin D supplementation and the risk of acute respiratory tract infection in the healthy pediatric group

**DOI:** 10.3389/fnut.2023.1188958

**Published:** 2023-06-20

**Authors:** Qiongyan Fang, Yingting Wu, Jie Lu, Huaiyu Zheng

**Affiliations:** ^1^Department of Pharmacy, Zhoushan Hospital of Zhejiang Province, Zhoushan, Zhejiang, China; ^2^Core Facilities Center of Capital Medical University, Beijing, China

**Keywords:** acute respiratory infections, vitamin D, randomized controlled trials, pediatrics, meta-analysis

## Abstract

No previous meta-analysis had explored the association between vitamin D supplementation in healthy pediatrics and the risk of acute respiratory tract infections (ARTIs). Thus, we meta-analyzed the current evidence in this regard to provide sufficient knowledge about this risk-benefit ratio for vitamin D supplementation in this specific age group. We searched seven databases for randomized controlled trials (RCTs) that investigated the effect of vitamin D supplementation and ARTIs risk on a healthy pediatric population (0–18 years old). Meta-analysis was performed through R software. We included eight RCTs after the screening of 326 records according to our eligibility criteria. There were comparable infection rates between Vitamin D and placebo groups (OR = 0.98, 95% CI = 0.90–1.08, *P*-value = 0.62), with no significant heterogeneity among the included studies (*I*^2^ = 32%; *P-*value = 0.22). Moreover, there was no significant difference between the two vitamin D regimens (OR = 0.85, 95% CI = 0.64–1.12, *P*-value = 0.32), with no considerable heterogeneity among the included studies (*I*^2^ = 37%; *P*-value = 0.21). However, there was a significant reduction in Influenza A rates in the high-dose vitamin D group compared to the low dose one (OR = 0.39, 95% CI = 0.26–0.59, *P-*value < 0.001), with no heterogeneity among the included studies (*I*^2^ = 0%; *P*-value = 0.72). Only two studies of 8,972 patients reported different side effects, with overall acceptable safety profile. Regardless of the dosing regimen used or the type of infection, in the healthy pediatric group, there is no evident benefit of using vitamin D to prevent or reduce the ARTI rates.

## 1. Introduction

The Acute respiratory tract infections (ARTIs) are a public health concern for both the clinical society and the patients. This concern stems from the significant morbidity and mortality rates, especially in winter. The burden is substantially high in low-income countries, where ARTIs contribute to 33% of all deaths in children below 5 years old (1, 2). Multiple risk factors were identified for inducing ARTIs, including young age, inadequate breastfeeding, and immunization, rural area, inadequate maternal nutrition, and malnutrition of paramount interest (1). Malnutrition is considered a primary comorbid condition with ARTIs, contributing to nearly one-fifth of patients with ARTIs (3).

Vitamin D is an essential component in the human body responsible for proper growth, skeletal health, and immunity. Research suggests that the seasonal variation of vitamin D plays a substantial role in the development of ARTIs where the vitamin D concentration drops significantly in the winter (4). The association between vitamin D levels and ARTIs was proven through a meta-analysis by Pham and colleagues (5). A recent meta-analysis by Jolliffe et al. (6) indicated that vitamin D was associated with decreased rates of ARI. However, the study results should be reported with some concerns as the authors combined several age groups (pediatric and adults) as well as participants' status (healthy and diseased) (6).

Recently, concerns were raised about whether vitamin D supplementation will be beneficial in reducing ARTIs, especially in the pediatric age groups. The intervention showed promising results by decreasing the rates of ARTIs hospitalizations and the incidence of ARTIs complications in pediatric patients with comorbidities (7, 8). However, the risk-benefit ratio should be weighted, especially when given as a protective agent in the healthy pediatric group. Evidence from many trials proved that vitamin D supplementation is ineffective in reducing ARTIs in healthy pediatrics (9–11). No previous meta-analysis had explored the association between vitamin D supplementation in healthy pediatrics and the risk of ARTIs. Thus, we will meta-analyze the current evidence in this regard to provide sufficient knowledge about this risk-benefit ratio for vitamin D supplementation in this specific age group.

## 2. Materials and methods

### 2.1. Search strategy and study selection

**Figure 1:**
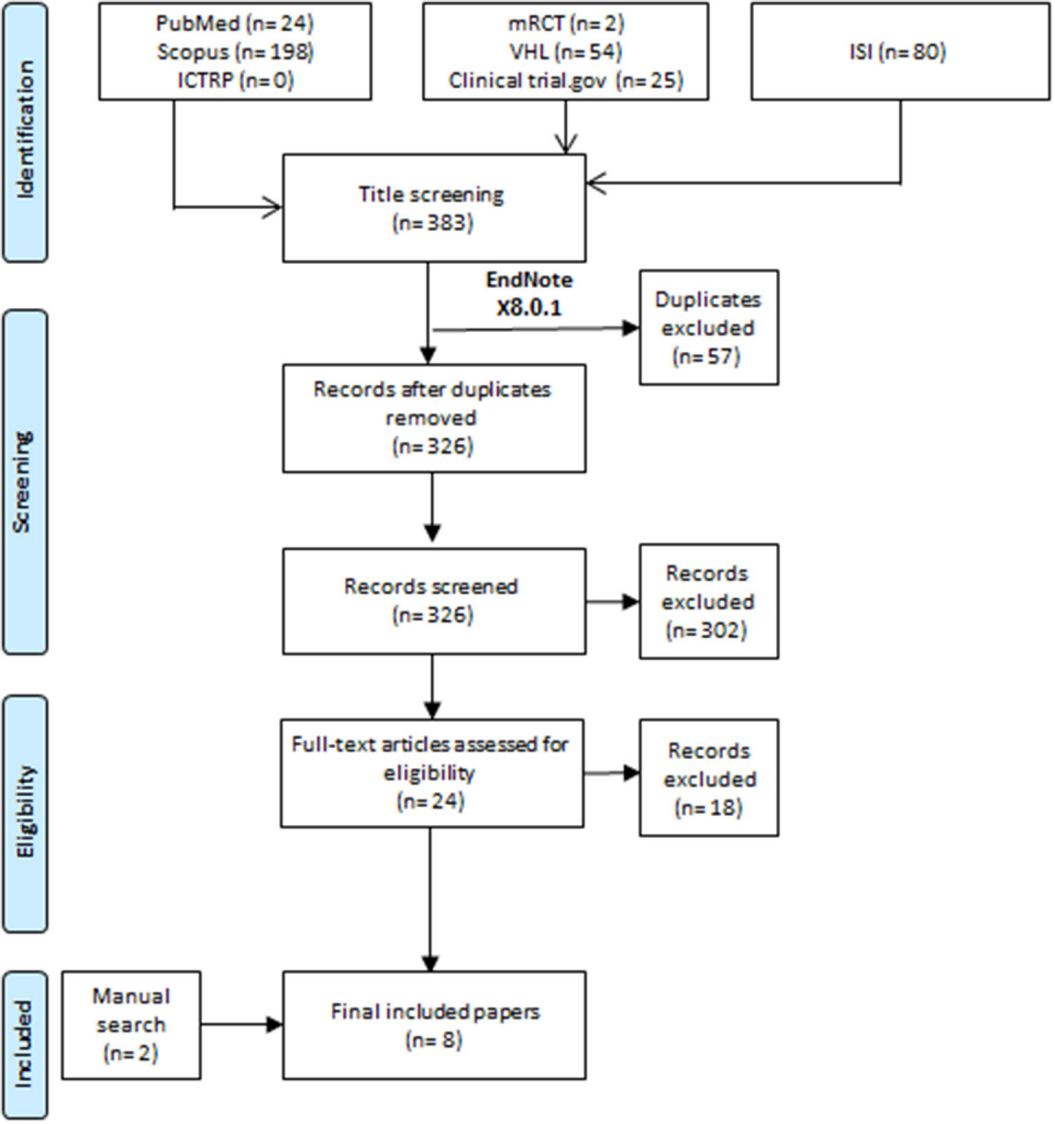
Flow diagram of the study process.

All the steps of this meta-analysis were conducted by following the Preferred Reporting Items for Systematic Review and Meta-analyses statement (PRISMA) recommendations (12). All authors shared in the keywords selection process for recruiting relevant articles. After the agreement between all authors, one author ran the search process on 27^th^ May 2022. The search process was conducted by transferring all records from seven databases (Figure 1) to Endnote software by using the search term “(“vit D” OR “vitamin D” OR “vitamin D”) AND (“acute respiratory infection” OR “ARI” OR “respiratory tract infection” OR “respiratory infection”) AND (children OR adolescent OR pediatric OR pediatrics) AND (trial OR RCT OR random OR randomized).” A process of removing duplicate records was conducted in the Endnote software before transferring the records to excel to start the process of title and abstract screening.

### 2.2. Eligibility criteria

We included randomized controlled trials (RCTs) that assessed the effect of vitamin D supplementation on healthy pediatric patients aged 0–18 years old on the risk of ARTIs. We excluded non-RCTs studies, conference papers, diseases pediatric participants aged > 18 years old.

The screening process followed our eligibility criteria and was done by three authors. Furthermore, the senior author developed an extraction sheet for extracting the relevant information. The sheet included characteristics (study ID, age, sex, country of patients, vitamin D dose, compared groups, and sample size) and outcomes (the prevalence of ARTIs and side effects of vitamin D supplementation). We used a cut-off value of 400IU/d as a standard dose of vitamin D, while we defined a high dose when the vitamin d supplementation exceeded 400IU/d. The screening, extraction, and quality assessment processes were done by two authors and revised by at least one author.

### 2.3. Risk of bias

The Well-known Cochrane risk of bias tool was used to rate the risk of bias in the included trials through three metrics (high risk, low risk or unclear risk).

### 2.4. Statistical analysis

One author used the R software version 4.0.2 to analyze the pooled data. The risk ratio and the corresponding 95% confidence intervals (CI) were used as an effect size for all the pooled outcomes using fixed or random effects. The model selection was chosen according to the level of heterogeneity at which we used a random model *I*^2^ value > 50% or *P*-value < 0.05 (13). *P*-value was considered significant if it was below 5%.

## 3. Results

### 3.1. Study characteristics

We screened 326 records according to our eligibility criteria, resulting in 28 full texts for further screening. Finally, we included six studies, and another two studies were included after the manual search of references of the included papers and any previously published systematic review (Figure 1) (9–11, 14–18).

Four studies compared high dose vitamin D supplementation vs. standard dose, and the other four compared vitamin D supplementation vs. placebo (Table 1). Two studies were conducted in Canada, and the rest in China, Mongolia, Finland, India, Vietnam, and Afghanistan. The follow-up ranged from 4 months to 3 years in all studies. Five studies were low risk of bias, while three were high risk of bias (Supplementary Figure 1).

**Table 1:** Characteristics of the included studies.

**Study ID**	**Country**	**Compared groups**	**Vit D dose**	**Sample size**	**Age [mean (SD)]**	**Male %**	**Follow up months**
Aglipay-2017	Canada	High dose vs. standard dose	2,000 IU/d−400 IU/d	349/354	2.7 (1.5)/2.7 (1.5)	55/60	6.2 months
Rosendahl-2018	Finland	High dose vs. standard dose	1,200 IU/d−400 IU/d	486/489	2 weeks−24 months^*^	50/51	24 months
Zhou-2018	China	High dose vs. standard dose	1,200 IU/d−400 IU/d	164/168	8 (2.7)/7.7(2.5)	51/53	4 months
Brett-2018	Canada	High dose vs. standard dose	600 IU/d−400 IU/d	24/25/25	2–8 years^*^	NR	1.5 months
Loeb-2018	Vietnam	Vit D vs. placebo	14,000 IU/d	650/650	8.6 (3.9)/8.4 (4)	50/46	12 months
Mandlik-2020	India	Vit D vs. placebo	1,000 IU/d	120/124	8.2 (1.2)/7.9 (1.1)	46/42	6 months
Holland-2012	Afghanistan	Vit D vs. placebo	100,000 IU/d	1,524/1,522	1–11 months^*^	53/51	18 months
Ganmaa-2020	Mongolia	Vit D vs. placebo	14,000 IU/d	4,418/4,433	9.4 (1.6)/9.4 (1.6)	51/50	3 years

* *Range, NR not reported*.

### 3.2. ARTIs

**Figure 2:**
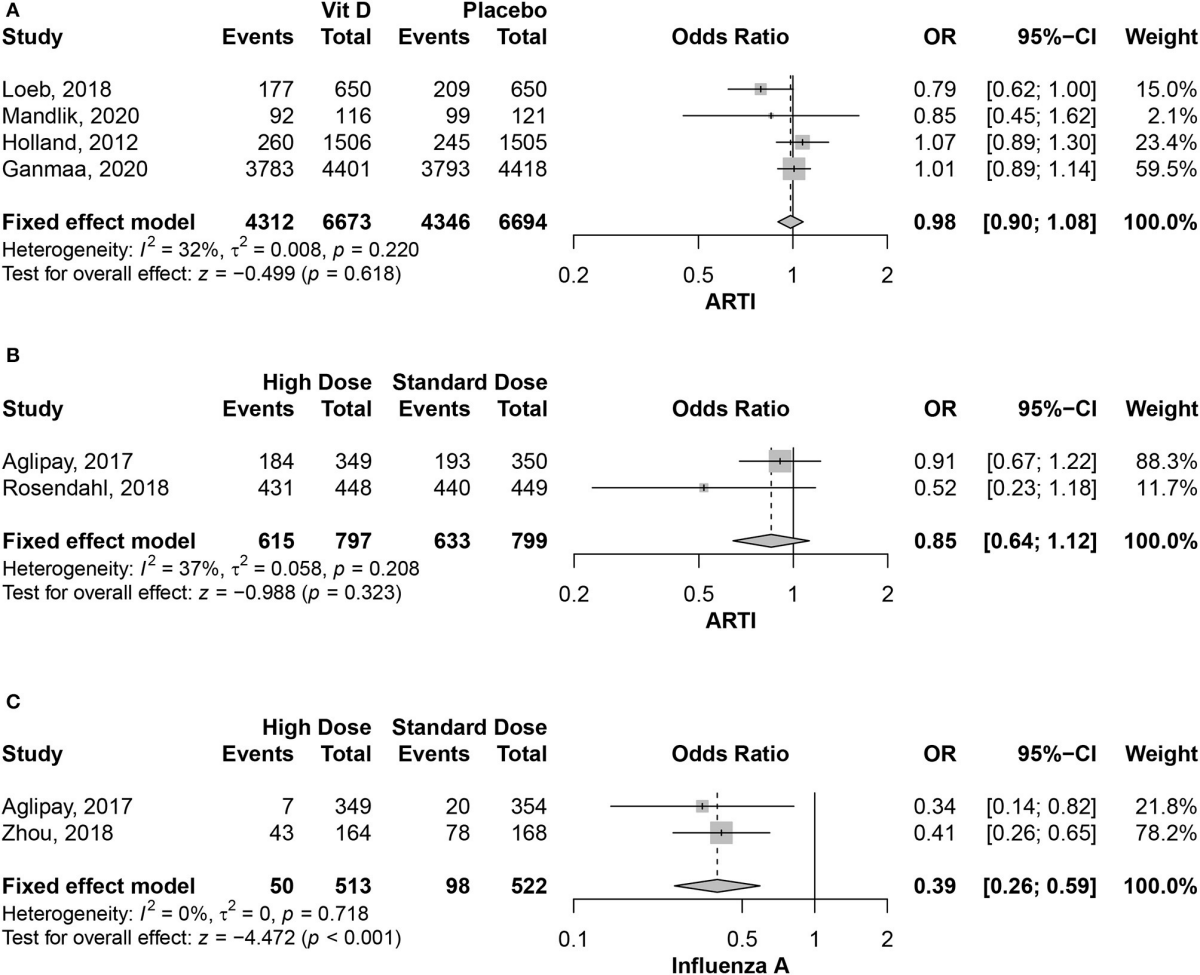
**(A)** Association between vitamin D supplementation and ARTIs. **(B)** Association between the different vitamin D supplementation and ARTIs. **(C)** Association between the different vitamin D supplementation and Influenza A virus.

The efficacy of vitamin D in preventing ARTIs was tested in four studies, with 13,367 individuals. There were comparable infection rates between Vitamin D and placebo groups (OR = 0.98, 95% CI = 0.90–1.08, *P*-value = 0.61), with no significant heterogeneity among the included studies (*I*^2^ = 32%; *P*-value = 0.22) (Figure 2A). In the same context, two studies, with 1,596 individuals, compared the high-dose vs. standard-dose of vitamin D in preventing ARTIs. There was no significant difference between the two vitamin D regimens (OR = 0.85, 95% CI = 0.64–1.12, *P*-value = 0.32), with no considerable heterogeneity among the included studies (*I*^2^ = 37%; *P*-value = 0.21) (Figure 2B).

Different studies investigated if ARTI prevention effect is variable across different causative viruses. Meta-analysis was possible in the Influenza A infection only, for being reported in more than one study using the treatment arms. There was a significant reduction in Influenza A rates in the high-dose vitamin D group compared to the low dose one (OR = 0.39, 95% CI = 0.26–0.59, *P*-value < 0.001), with no heterogeneity among the included studies (*I*^2^ = 0%; *P*-value = 0.72) (Figure 2C).

**Figure 3:**
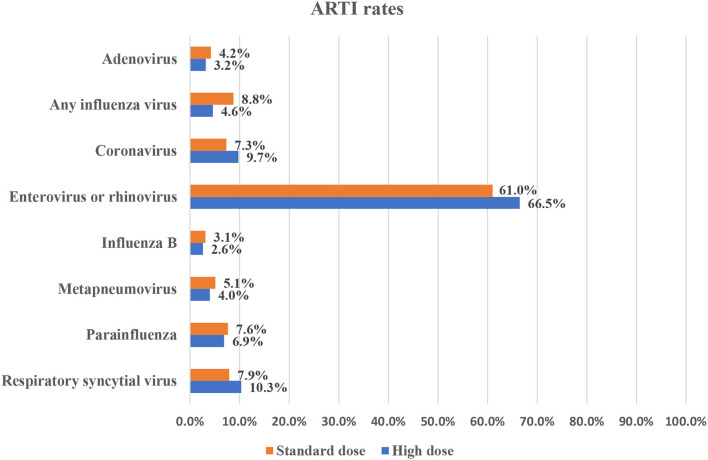
Infection rates in the different vitamin D supplementation.

**Figure 4:**
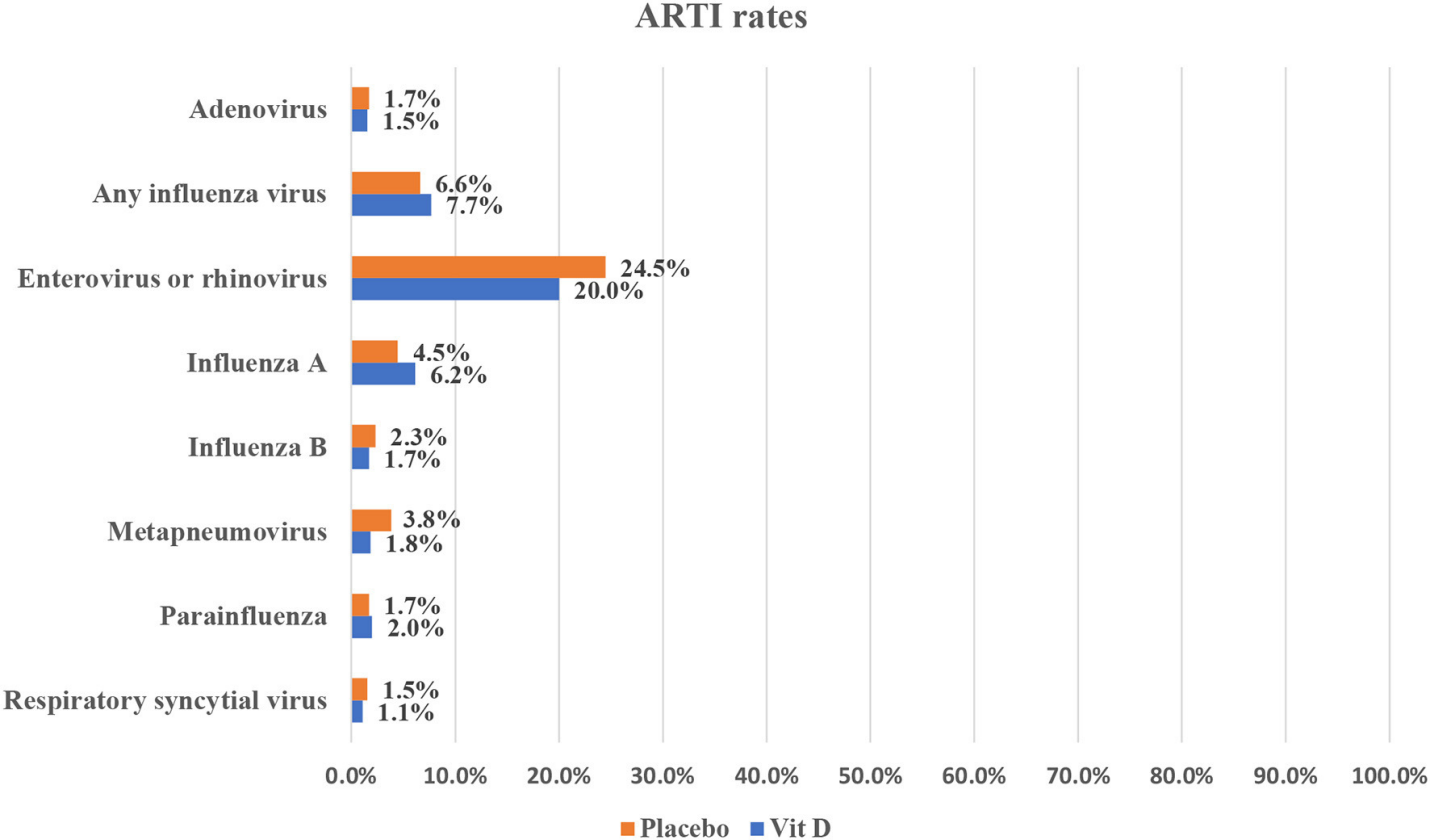
Infection rates in vitamin D supplementation vs. placebo.

For all other infections, they were reported in only one study each with the same comparison groups. The rates of different infections as reported in the included studies are summarized in Figures 3, 4.

Only two studies of 8,972 patients reported different side effects (16, 18). Ganmaa et al. (18) reported the death of 10 children (four in vitamin D group vs. six in the placebo one) and 324 children had serious adverse events (142 in vitamin D group vs. 182 in the placebo one); however, according to the authors, none of them were related to the participants' allocation in either group. Only one symptomatic hypercalcemia (nausea and epigastric pain) was reported in the vitamin D group and none in the placebo one. Furthermore, three children had hypervitaminosis D with no symptoms, and none had renal stones in either group. Zhou et al. (16) reported a good safety profile with no circulatory, hematologic, renal, or nervous symptoms among all infants. Nevertheless, four infants (two in high-dose vitamin D and two in low-dose vitamin D) showed possible poisoning symptoms (diarrhea and/or vomiting), which were deemed to be induced by gastrointestinal infection, not vitamin D.

## 4. Discussion

The current study provides collective evidence on the association between vitamin D supplementation and ARTI risk in the pediatric group. Based on our findings, vitamin D has no evident benefit in significantly reducing ARTI rates compared to giving a placebo. Moreover, using a high dose of vitamin D did not have any added value compared to the standard dose regimen. Furthermore, the rates of different viral infections were comparable, regardless of the regimen or causative organism, in almost all infections reported; however, meta-analysis was not possible to provide pooled estimates. However, using the high dose regimen of vitamin D showed a significant reduction in Influenza A rates compared to the low dose one.

The current literature has different reports linking the low blood 25(OH)D levels and the higher risk of ARTI in the general population and pediatric groups (19–24). The biological effects of vitamin D are primarily mediated through the vitamin D receptors, which are abundant throughout the human body (25). Blood levels of 25(OH)D have been linked to developing adaptive and innate immunity responses, with enhanced production of antimicrobial peptides, regulating immune cell proliferation, maturation, and cytokine expression, as early as the first 6 months of life (26–28). It has also been reported that the lower cord serum level of 25(OH)D is associated with impaired lung function in infants (28), which may provide an explanation of the vitamin D involvement in affecting ARTI susceptibility. It should be noted that temperature and ultraviolet rates could play role in the development of influenza infection which reaches it is peak in low temperature in addition to low sun exposure (29). However, this observation was not taken in consideration in our results due to the scarcity of data among all the included papers.

Vitamin D supplementation's efficacy in reducing these infections was investigated; however, the results were inconsistent among different studies. A trial comparing the efficacy of a daily dose of 800 IU of vitamin D to a placebo, found a reduction in primary care visits for ARTI for infants using this regimen from birth to 6 months compared to those given the placebo (2). Another larger trial of a quarterly bolus dose of vitamin D to prevent pneumonia infection among infants found no obvious benefit of this regimen in reducing pneumonia incidence (30). In the same context, trials have found that children with baseline deficiency or insufficiency of vitamin D would benefit from vitamin D supplementation as a protective intervention against ARTI (11, 31). A previous meta-analysis in the general population found a beneficial effect of daily or weekly doses of vitamin D to prevent ARTI in individuals with severe vitamin D at baseline (9). These discrepancies in results may be explained by the heterogeneous sample sizes, age groups, dosing regimens, and the baseline vitamin D status of the study population.

Although this is the first meta-analysis of randomized controlled trials on this topic, it has some limitations. First, different treatment arms were compared in the included studies, preventing the pooling of all studies within the same analysis. Second, stratifying vitamin D efficacy by the infection type was heterogeneous and infrequent, preventing the synthesis of a collective evidence per infection type. Thirdly, we could not study the effect of seasonal variation, sun exposure and other related factors that could play a significant role in changing the vitamin D level, due to the scarcity of data among the included papers. Finally, the safety profile was discussed in only two studies, which is an important piece of evidence needed to provide a comprehensive assessment.

## Data availability statement

The original contributions presented in the study are included in the article/Supplementary material, further inquiries can be directed to the corresponding author.

## Author contributions

QF and HZ conceptualized and contributed to methodology. QF performed software analyses while YW performed data validation. QF, JL, and HZ did formal analysis. JL contributed to data curation and writing of the original draft. QF and YW reviewed and edited the manuscript while HZ supervised the whole study. All authors have read and agreed to the published version of the manuscript.
