# Supplementary material for: A meta-analysis of the association between vitamin D supplementation and the risk of acute respiratory tract infection in the healthy pediatric group
Source: Front Nutr. 2023 Jun 20;10:1188958. doi: 10.3389/fnut.2023.1188958 (PMC10318162; doi:10.3389/fnut.2023.1188958)
Supplement: Supplementary file 1 [file Data_Sheet_1.docx]

Supplementary Material

A meta-analysis of the association between vitamin D supplementation and the risk of acute respiratory tract infection in the healthy pediatric group

Qiongyan Fang^1^, Yingting Wu^2^, Jie Lu^1^, Huaiyu Zheng^1^*,

*** Correspondence:** Huaiyu Zheng: zhenghuaiyu8@outlook.com


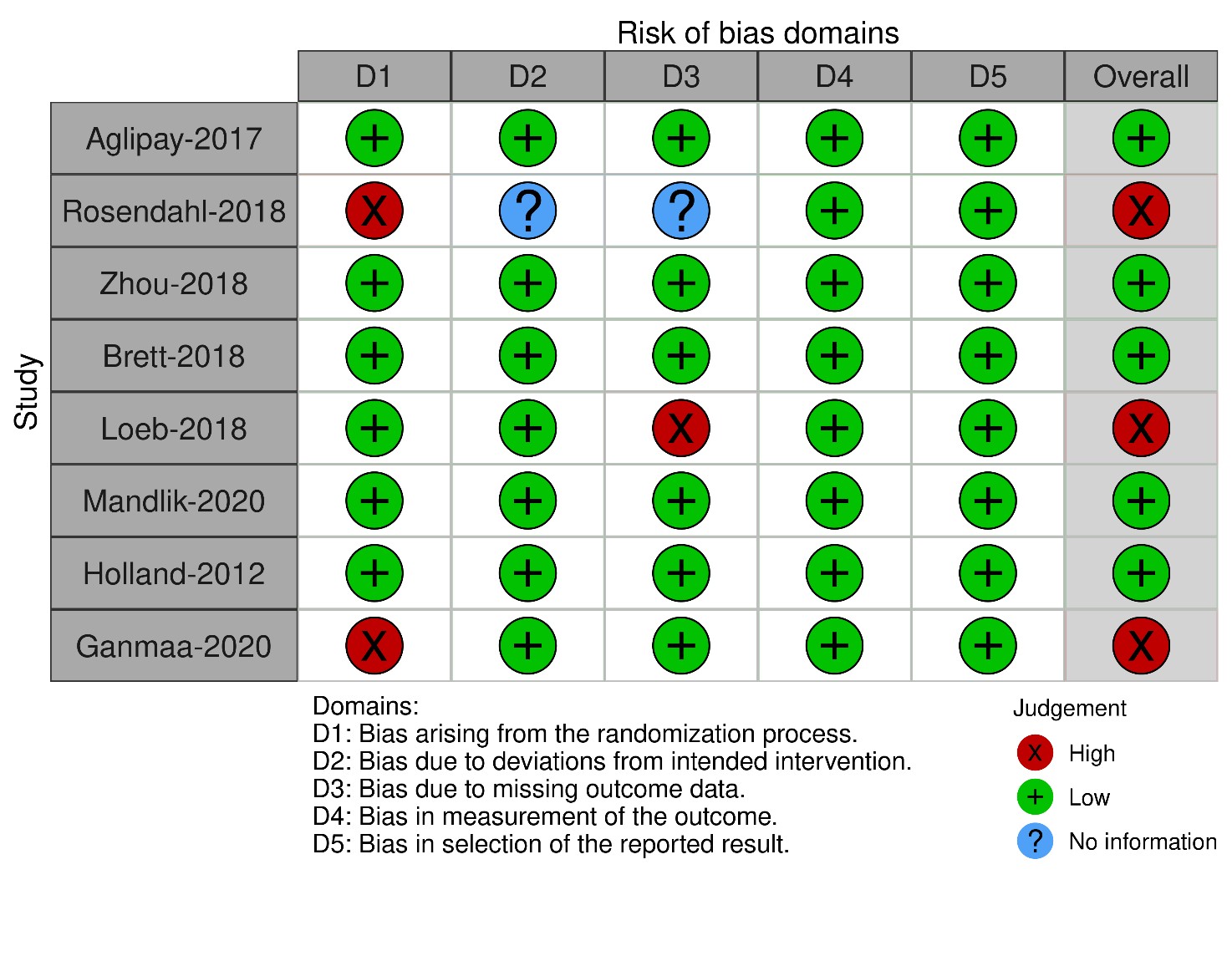


**Supplementary Figure 1.** Studies comparing the risk of bias domains.
